# Phenotypic, genotypic and biochemical changes during pyrethroid resistance selection in *Anopheles gambiae* mosquitoes

**DOI:** 10.1038/s41598-020-75865-1

**Published:** 2020-11-04

**Authors:** Maxwell G. Machani, Eric Ochomo, Daibin Zhong, Guofa Zhou, Xiaoming Wang, Andrew K. Githeko, Guiyun Yan, Yaw A. Afrane

**Affiliations:** 1 Entomology Section, Centre for Global Health Research, Kenya Medical Research Institute, Kisumu, Kenya; 2 School of Health Sciences, Jaramogi Oginga Odinga University, Kisumu, Kenya; 3 Program in Public Health, College of Health Sciences, University of California, Irvine, CA 92697 USA; 4 Department of Medical Microbiology, University of Ghana Medical School, College of Health Sciences, University of Ghana, Accra, Ghana

**Keywords:** Entomology, Zoology

## Abstract

The directional selection for insecticide resistance due to indiscriminate use of insecticides in public health and agricultural system favors an increase in the frequency of insecticide-resistant alleles in the natural populations. Similarly, removal of selection pressure generally leads to decay in resistance. Past investigations on the emergence of insecticide resistance in mosquitoes mostly relied on field survey of resistance in vector populations that typically had a complex history of exposure to various public health and agricultural pest control insecticides in nature, and thus the effect of specific insecticides on rate of resistance emergency or resistance decay rate is not known. This study examined the phenotypic, genotypic, and biochemical changes that had occurred during the process of selection for pyrethroid resistance in *Anopheles gambiae*, the most important malaria vector in Africa. In parallel, we also examined these changes in resistant populations when there is no selection pressure applied. Through repeated deltamethrin selection in adult mosquitoes from a field population collected in western Kenya for 12 generations, we obtained three independent and highly pyrethroid-resistant *An. gambiae* populations. Three susceptible populations from the same parental population were generated by removing selection pressure. These two lines of mosquito populations differed significantly in monooxygenase and beta-esterase activities, but not in *Vgsc* gene mutation frequency, suggesting metabolic detoxification mechanism plays a major role in generating moderate-intensity resistance or high-intensity resistance. Pre-exposure to the synergist piperonyl butoxide restored the susceptibility to insecticide among the highly resistant mosquitoes, confirming the role of monooxygenases in pyrethroid resistance. The rate of resistance decay to become fully susceptible from moderate-intensity resistance took 15 generations, supporting at least 2-years interval is needed when the rotational use of insecticides with different modes of action is considered for resistance management.

## Introduction

The directional selection for insecticide resistance due to indiscriminate use of insecticides in public health and agricultural system favors an increase in the frequency of insecticide-resistant alleles in the natural populations^1^. The emergence of insecticide resistance not only shortens the lifespan of the existing vector control tools but also undermines the efficacy of novel developed vector control products through cross-resistance or multiple resistance^2,3^. In response to this challenge, the Global Plan for Insecticide Resistance Management (GPIRM)^4^ of the World Health Organization (WHO) came up with strategies to preserve the effectiveness of current vector control tools and at the same time develop new and innovative vector control tools for the future, to significantly reduce malaria morbidity and mortalities. In the face of the current challenge, knowledge on the molecular changes and evolutionary process driving insecticide resistance in resistant populations is crucial toward more effective resistance management. Past investigations on the emergence of insecticide resistance in mosquitoes relied on field survey of resistance in vector populations that typically had a complex history of exposure to various types of insecticides^5–7^ and thus the effect of specific insecticide selection pressure on the rate of resistance emergency is not known.

Resistance to pyrethroids, the most commonly used insecticide for malaria vector control worldwide has been linked to the two mechanisms: target site insensitivity and increased detoxification^8^. Target-site insensitivity is caused by point mutations in the voltage-gated sodium channel (*Vgsc*) gene targeted by pyrethroids. In *Anopheles gambiae,* the most important malaria vector in Africa, 1014S and 1014F mutations in *Vgsc* confer resistance to pyrethroids and DDT, and the frequency of these mutations is near fixation in the majority of natural populations in Africa^9–12^. The metabolic detoxification caused by increased activity of detoxification enzymes that metabolize insecticides before reaching the target site has been linked to elevated levels of P450 monooxygenase enzymes^13,14^. Other resistance mechanisms such as cuticular thickening and overexpression of chemosensory proteins have been reported^15,16^. Increased resistance in malaria vectors has been associated with the intensive use of pyrethroids-treated nets and indoor residual spraying^9,11,17,18^. On the other hand, a number of studies have reported the indiscriminate use of other classes of insecticides for agricultural purposes added selection pressure for resistance in mosquito populations^2,5,7^.

Resistant insects may have an adaptive advantage over susceptible insects in an environment where there is a continuous pressure due to insecticide use. However, this fitness advantage may decrease over time in the absence of treatments^19^. The extent to which a resistant population is exposed to insecticides may have a strong impact on whether resistance can persist or even decrease in the population. One important insecticide-resistant management strategy recommended by WHO is the rotation and mixture of insecticides of different modes of action to retard or reverse the spread of resistance^4^. However, the frequency of rotation of different insecticides is unknown, and one key determinant is the rate of resistance decay after removal of selection pressure. To date, few studies have directly investigated changes of insecticide resistance upon removal of insecticide selection pressure. In this context, understanding the evolution of resistant mosquitoes and tracking changes in resistance under different environments over time will greatly improve the resistance management strategies. This study sought to better understand the process of insecticide resistance development, or resistance decay in the absence of selection pressure, in *An. gambiae* mosquitoes.

## Methods

### Mosquito population and strain used

**Figure 1:**
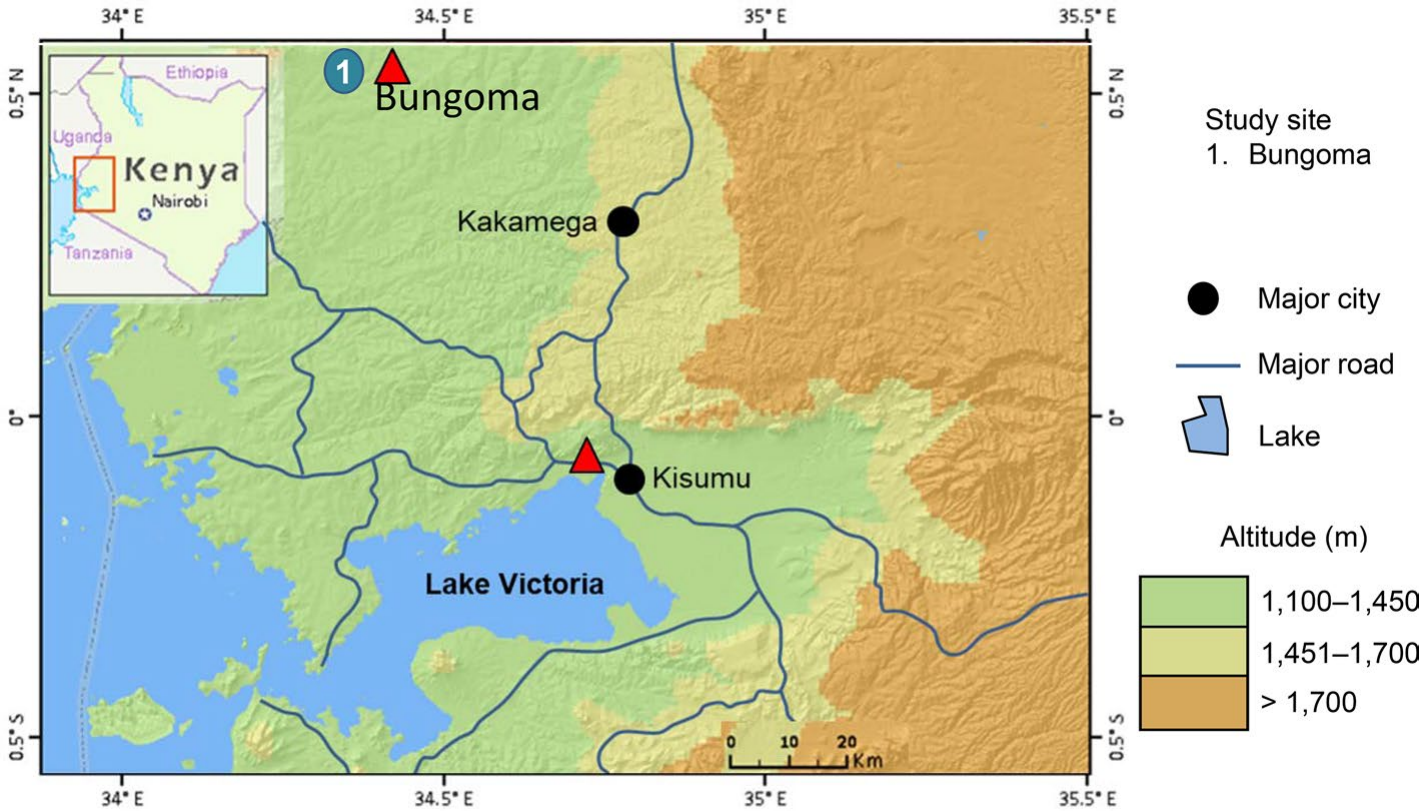
Map showing mosquito collection site in western Kenya.

Selection of *An. gambiae* for pyrethroid resistance was undertaken with mosquitoes collected from Bungoma county (00.590531°N, 034.388066°E, altitude 1545 m above the sea level) in western Kenya (Fig. 1). Blood fed females were collected from the field using mouth aspirators, and then transported to the insectary of the Centre for Global Health Research, Kenya Medical Research Institute (KEMRI) in Kisumu, western Kenya. Mosquito collections took place in several villages in Bungoma over a 6-week period to avoid collecting vectors from the same mothers. Gravid females were allowed to lay eggs in individual ovipositor cups, and they were confirmed for their species identity using PCR^20,21^. Egg batches were transferred to individual paper cups containing rainwater for hatching in the insectary. *An. gambiae* s.s F_1_ larvae were pooled and fed on Tetramin baby fish food and brewer’s yeast daily, and *An. arabiensis* larvae were discarded. Larvae were reared under standard conditions (26 ± 2 °C; 80% ± 10% relative humidity (RH) with 12 h: 12 h light/dark cycle). Upon pupation, individuals were collected and transferred to cages and allowed to emerge as adults. From the day of emergence, adults were provided with cotton wool soaked with a 10% sugar solution until ready to be used for bioassay tests. The Kisumu reference strain which has been colonized since 1953^22^ and free of any detectable insecticide resistance mechanism was maintained under the same insectary conditions and used as a reference susceptible strain in all bioassays.

### Pyrethroid selection

F_1_ female adults that emerged were separated into two groups: the selected group (R group) for increased resistance and the unselected group (S Group) to determine the rate of resistance decay in the absence of selection pressure. For the R group, newly-emerged 3–5 days old F_1_ adult females from the field collection were exposed to 0.05% deltamethrin treated papers for 1 h according to the standard WHO susceptibility tube test protocol in batches of 25 mosquitoes with four replicates^23^. After each test, females were transferred to recovery cups and provided with a 10% sugar solution on cotton wool and held for 24 h at 26 ± 2 °C and 80 ± 10% °C RH under L12: D12 h light: dark cycle. Mortality was recorded 24 h after the test, and surviving females were then transferred to cages for blood feeding and egg-laying. In the following generations, adult female mosquitoes were subjected to the same selection procedure. Population size used to found each generation was over 200 mosquitoes for any lineages to minimize the effect of founder effect. To determine the reproducibility of the selection experiment, three independent lineages, named R1, R2 and R3 were maintained. For the S Group, 3 distinct lineages named S1, S2 and S3 were created and reared under the same conditions as the R Group except that there was no insecticide selection. This process was repeated for a total of 12 generations for the R Group and 17 generations for the S Group. Due to the low population numbers, selection was not conducted for generations 8 and 9 for the R Group. Insecticide resistance was determined using the WHO susceptibility tube test at every generation. The R Group and S Group populations were maintained in parallel, but generation difference between the two groups was caused by longer larva-to-pupa development time of the R Group. From the selected population (F_3_ and F_6_) and unselected population (F_9_ and F_13_) were chosen for specific assays based on the largest observed difference in phenotypic resistance when compared to the parent population.

### Insecticide resistance intensity assays

Resistance intensity bioassays were conducted using 5× and 10× diagnostic concentrations of deltamethrin papers to enable better characterization of resistance intensity in the R Group. The test was identical to the standard WHO insecticide susceptibility tube test except that 5× and 10× deltamethrin concentrations were used in the test papers. Four replicates each containing 25 female adult mosquitoes aged 3–5 days from parental population and F6 of the R Group were used for resistance intensity bioassays.

### Piperonyl butoxide synergist tests

To understand the role of metabolic detoxification in pyrethroid resistance, piperonyl butoxide (PBO), a synergist that inhibits the specific activity of P450 monooxygenases in insects was used in the resistance bioassay. Briefly, unfed females aged 3–5 days were pre-exposed to 4% PBO impregnated test papers for 1 h, and then immediately exposed to 0.05% deltamethrin for another hour. One batch of 25 females was only exposed to 4% PBO without insecticide as a control. Mosquitoes were transferred to holding tubes and supplied with 10% sugar solution. Mortality was scored after the 24 h recovery period. Mosquitos from the parental population and F_6_ generation of the R Group were tested.

### Mutation analysis of the voltage-gated sodium channel (*Vgsc*) and acetylcholinesterase-1 (*Ace*-1) genes

DNA was extracted from the legs of adult female mosquitoes as previously described^20^. Taqman assays was used to detect mutations at amino acid position 1014 and 1575 of *Vgsc*^24,25^. Mosquitoes randomly selected from parent population, F3 of the R Group, and F9 and F13 of the S group were genotyped. The same set of samples were also genotyped for G119S mutation in *Ace*-1^26^.

### Metabolic enzyme activity assay

Selected mosquitoes were tested for activities of three detoxification enzymes, monooxygenases, beta (β) esterases and glutathione *S*-transferases (GSTs)^27,28^. Individual mosquitoes were homogenized in a 1.5-ml tube with 300 μl of 0.25 M phosphate buffer (pH 7.2) and diluted by adding 300 μl of phosphate buffer. The content of the tube was mixed and centrifuged, and the supernatant was used to test enzyme activities. All assays were carried out in triplicate, and the protein content of the supernatant was measured using the Bradford method^27^. For GSTs, a total of 90 μl of reduced glutathione solution and 90 μl of 1-chloro-2,4′-dinitrobenzene (cDNB) solution was added to 90 μl of mosquito supernatant. The absorbance was measured immediately using a microplate reader at 340 nm and then detected every 2 min for five times, using 0.25 M phosphate buffer as the negative control. For monooxygenases, a total of 200 μl of 3,3–5,5-tetramethyl-benzidine dihydrochloride hydrate solution was added to 100 μl of mosquito supernatant. A volume of 25 μL of 3% hydrogen peroxide was then added and incubated for 5 min, absorbance was read at 620 nm. For β-esterases, a total of 100 μl of β-naphthyl acetate solution was added to 100 μl mosquito supernatant and incubated at room temperature for 10 min. a total of 100 μl of dianisidine (100 mg/100 mL water) was added. The sample absorbance was read at 540 nm. Samples from the parent population, F3 and F6 of the R Group, F13 of the S Group, and susceptible Kisumu reference strain were examined.

### Statistical analysis

WHO (2016) criteria were used to classify the resistance or susceptibility status of the tested mosquito populations^29^. For the WHO resistance intensity bioassay, mortality of < 98% at 5× concentration indicates moderate to high-intensity resistance, and mortality between 98–100% at 5× concentration indicates low-intensity resistance. Mortality between 98–100% at 10× concentration confirms moderate-intensity resistance and mortality of < 98% at 10× concentration indicates high-intensity resistance. Analysis of variance (ANOVA) was used to determine among-population differences in mosquito mortality rate in the insecticide susceptibility bioassay^29^. The *Vgsc* allele frequency was calculated for each population at different generations and statistical differences among populations were examined using the F exact test. ANOVA also was used to examine whether activities of monooxygenases, esterases and GSTs varied significantly among populations and over generation. The analysis was done using open source software, R programming language^30^.

## Results

### Pyrethroid resistance changes during the selection process

**Figure 2:**
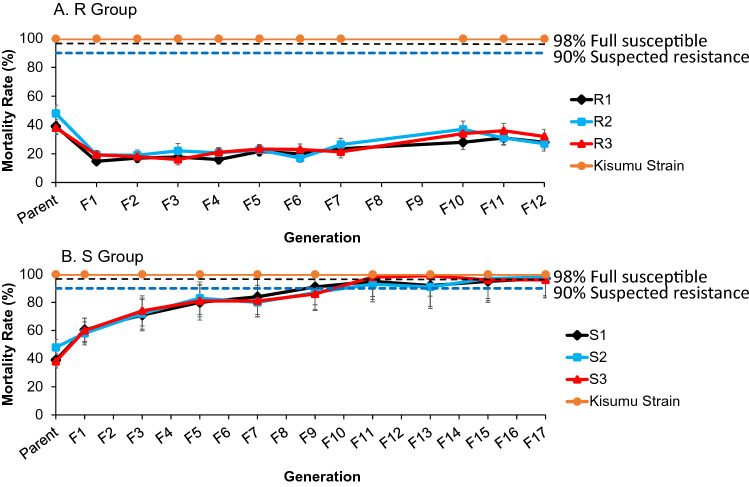
Dynamics of mortality rates of the selected pyrethroid resistant *Anopheles gambiae* line (top) and susceptible line (bottom). Mortality rate was measured using the WHO insecticide susceptibly tube bioassay for deltamethrin. Three independent populations were generated for the resistant line (R1, R2 and R3) and susceptible line (S1, S2 and S3). Error bars indicate 95% confidence intervals. The 90% mortality threshold for declaring suspected resistance and 98% mortality threshold for calling full susceptibility based on the WHO criteria are indicated.

The mortality of the WHO susceptibility tube bioassay in the parent populations averaged 42% (ranging from 38 to 48% for the 3 lineages). After one generation of selection, resistance was significantly increased, with mortality rate to 18% (ranging from 15 to 19%) (P < 0.001; Fig. 2A). Resistance was maintained when the mosquito populations were subjected to pyrethroid selection. However, an increase in mortality rate was observed (mortality rate; averaged 33%) in F10 when selection pressure was lifted for F8 and F9 mosquitoes in the R Group. The three independent lineages in the R Group exhibited a remarkably similar trend in the resistance (Fig. 2A).

The populations without insecticide selection pressure became progressively more susceptible over the course of 18 generations (Fig. 2B). Based on WHO criteria, the parent populations were classified as highly resistant. After 9 generations without selection pressure, the populations were reverted to the status of “suspected resistance”, and after 15 generations the populations became fully susceptible. Overall, the three independent lineages in the S Group exhibited a very similar trend in the loss of resistance.

### Resistance intensity bioassay

**Figure 3:**
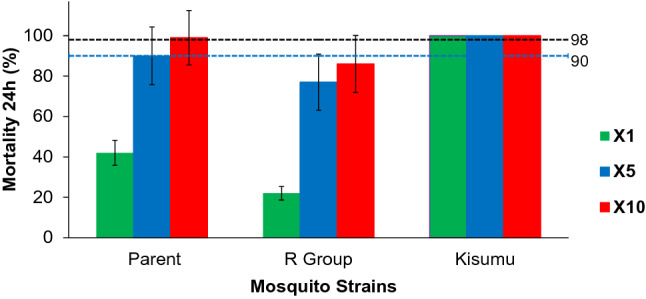
Insecticide intensity bioassay results. Standard resistance diagnostic concentration (0.05% deltamethrin, or 1×), 5× and 10× concentrations of deltamethrin were used WHO insecticide susceptibly tube bioassay. Kisumu strain is the standard WHO susceptible reference population. Error bars indicate 95% confidence intervals. The 90% mortality threshold for declaring suspected resistance and 98% mortality threshold for calling full susceptibility based on the WHO criteria are indicated.

The mortality of the parent population exposed to 1×, 5× and 10× of the diagnostic doses of deltamethrin were 42%, 90% and 99% respectively, indicating “moderate-intensity resistance” by the WHO criteria. The mortality for F6 from the R Group exposed to 5× and 10× diagnostic doses of deltamethrin was 77% and 86% respectively (Fig. 3), indicating the F6 generation of the R Group reached “high-intensity resistance”. One hundred percent mortality was recorded for the Kisumu susceptible reference strain, confirming the quality of the insecticide-treated papers used and bioassay test.

### PBO synergist bioassay

**Figure 4:**
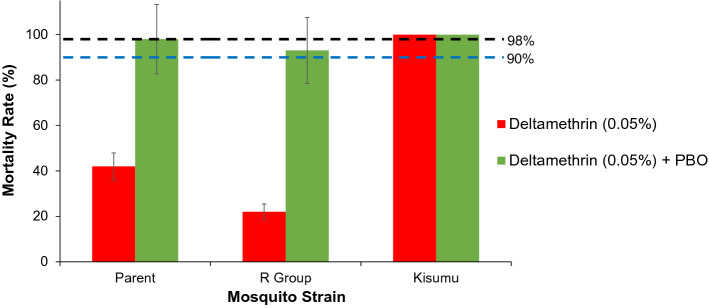
Effect of pre-exposure to synergist piperonyl butoxide (PBO) on insecticide susceptibility restoration *Anopheles gambiae* populations. Mortality rates of the WHO insecticide susceptibly tube bioassay was shown. The “parent” population belonged to moderate intensity resistant, and R Group mosquitoes were in high intensity resistance for deltamethrin. Error bars indicate 95% confidence intervals. The 90% mortality threshold for declaring suspected resistance and 98% mortality threshold for calling full susceptibility based on the WHO criteria are indicated.

Pre-exposure of the parent population to PBO before exposure to deltamethrin increased mortality rate from 42 to 98% (P < 0.001; Fig. 4), suggesting that PBO restored mosquito susceptibility to deltamethrin. Similarly, the synergistic effect of PBO was large in F6 of the R Group as evidenced by a significant increase in WHO tube bioassay mortality rate from 22 to 95% (P < 0.001), suggesting that monooxygenases played an important role in deltamethrin resistance both in the field populations and in the laboratory selected population of high-intensity resistance (Fig. 4).

### Frequency of *Vgsc* and *Ace*-1 mutations

The frequency of *Vgsc* wildtype allele L1014 was low (0.03), and the two mutations causing resistance (L1014S and L1014F) reached fixation (Table 1). The L1014 allele was lost in R Group mosquitoes, and decreased insignificantly in the S Group mosquitoes. The R Group and S Group mosquitoes differed significantly in the frequency of L1014F and L1014S (Table 1). The 1575Y of *Vgsc* and *Ace*-1 mutations were not detected in any of the populations tested.

**Table 1:** Allele frequency of *Vgsc* and *Ac*e-1 genes in populations under different insecticide selection schemes.

Population	Generation	Sample size	*Vgsc*				*Ace*-1
			Locus 1014			Locus 1575	Locus 119
			L1014	L1014S	L1014F	1575Y	G119S
Parent population		196	0.03	0.88	0.09	0	0
R Group	F3	179	0	0.77	0.23	Not done	0
S Group	F9	176	0.01	0.98	0.01	Not done	0
S Group	F13	176	0.02	0.98	0.00	Not done	0

*F* filial generation, *R* selected strain, *S* unselected strain.

### Metabolic enzyme assay

**Figure 5:**
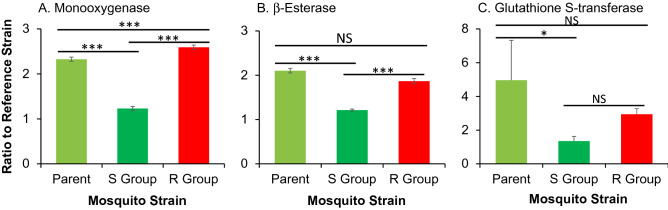
Effect of selection for deltamethrin resistance on the activities of three metabolic detoxification enzymes in *Anopheles gambiae*. **(A)** Monooxygenases; **(B)** β-esterases; and **(C)** glutathione *S*-transferase. Enzyme activities were expressed as the ratio of a population of interest to the Kisumu reference strain. Error bars indicates 95% confidence intervals. *, P < 0.05; ***, *P* < 0.001; *NS* not significant.

The monooxygenases, β-esterases and GST activity were analysed to determine if there were any changes during the selection process of resistance. The result showed that the monooxygenase activities were significantly reduced when the populations were not exposed to insecticide selection pressure, by 46.9% (P < 0001; Fig. 5A). Monooxygenase activities were increased by 11.4% in the selected resistant group (P < 0.001). For β-esterases, the unselected population (S Group) exhibited 42.3% reduction from the parent population (P < 0.001), and selected resistant population (R Group) showed non-significant changes from the parent population (Fig. 5B). Similar results were found for GST, in which the S Group showed 72.8% reduction from the parent population (P < 0.001), but non-significant changes in the R group (Fig. 4C). Overall, unselected S Group and selected R group exhibited significant divergence in monooxygenase and β-esterase activities, but not in GST activities (Fig. 5). Significant increase in monooxygenase activities in the Selected R Group and significant decrease in monooxygenase, β-esterase and GST activities in the unselected S Group suggest that monooxygenases play the most important role and β-esterases and GSTs less important role in pyrethorid resistance.

## Discussion

Insecticide resistance monitoring and mechanistic studies are key to insecticide resistance management. Current insecticide-resistant management strategies of WHO include rotational and a mixture of insecticides to retard or reverse the spread of resistance^4^, with the assumption that resistant phenotypes will be less fit in the absence of insecticide compared with susceptible. The results of this study show that under continuous insecticide selection pressure a high level of deltamethrin resistance emerged and was maintained in *An. gambiae*. However, in the absence of insecticide, highly resistant *An. gambiae* population was progressively reverted to be susceptible after 15 generations, or about 1.3 years in typical malaria-endemic African settings^31–33^. Although the study reported in this paper was conducted under the laboratory conditions with one most commonly used insecticide in Africa, our data on the rate of resistance decay when insecticide selection pressure is released has implications on the strategy of malaria vector resistance management through rotation of different insecticides. The highly resistant and susceptible mosquito populations that descended from the same genetic background we obtained from this endeavor also offer laboratory colonies for future behavioral and molecular studies of insecticide resistance.

Examples of insecticide resistance instability in the absence of insecticide in semi-field and laboratory conditions were reported in *Aedes aegypti* and *Culex pipiens* mosquitoes^31,34,35^. The loss of resistance was evident in our selected resistance population (R Group) in which mortality rate of the WHO insecticide susceptibility test was increased from 23% in F7 to 33% in F10 when selection pressure was not imposed in F8 and F9. This finding suggests that resistance under field conditions can diminish without insecticide pressure, probably due to fitness costs associated with insecticide resistance in the absence of insecticide selection^36^. This phenomenon was similar to cotton bollworm (*Helicoverpa armigera*) in the field in Benin, West Africa where the level of insecticide resistance increased quickly during the application of insecticides and decreased when insecticide treatment was suspended^37^.

The intensity resistance bioassay used in this study enabled us to further classify resistance to moderate-intensity resistance” or “high-intensity resistance”. In the parent population collected from the field, pyrethroid resistance was in the status of moderate-intensity resistance. After six generations of selection, the population was elevated to high-intensity resistance. The selection pressure we used in the laboratory was perhaps more stringent than the natural situation, but high intensity resistance *An. gambiae* were reported in natural populations from Ghana west Africa^38^. Such elevated high-intensity resistance resulted completely from metabolic resistance, but not from the *Vgsc* target-site insensitivity mechanism. *Vgsc* gene mutation frequencies did not differ between the selected resistant populations (R Group) and unselected susceptible populations (S Group). The unselected susceptible populations did not exhibit significant decrease in allele frequency of *Vgsc* mutations (L1014S and L1014F), suggesting that mutations at *Vgsc* played little role in pyrethroid resistance in the mosquito populations we studied. On the other hand, pre-exposure to synergist PBO dramatically reduced resistance in the selected resistant population with moderate resistance intensity (R Group) and rendered the field collected resistant population (Parent) to be fully susceptible. The results of the PBO synergist bioassay and metabolic enzyme analyses suggest that pyrethroid resistance observed was primarily mediated by increased activity of monooxygenases.

Our findings on the effect of PBO synergist is consistent with previously published findings in *An. gambiae*^13^ and in *Culex pipiens*^30^. PBO is known to enhance the effects of insecticides in resistant insects by inhibiting monooxygenases metabolic enzyme activities^39^. The restoration of susceptibility in a population showing high resistance with the use of PBO demonstrates that the incorporation of synergists with pyrethroids may be useful in restoring its effectiveness against resistant mosquitoes. Considering the current worldwide use of pyrethroids, there is a tremendous selection pressure for increased resistance in the mosquitoes. The use of synergists offers a temporary solution for maintaining the effectiveness of pyrethroid-based vector control tools, but the long-term solution of insecticide resistance management relies on integrated vector control strategy with minimal use of chemical insecticides.

## Conclusion

We obtained three highly pyrethroid-resistant *An. gambiae* populations through repeated deltamethrin selection. Three susceptible populations from the same genetic background were generated simply by removing selection pressure. These two lines of mosquito population differed significantly in monooxygenase and β-esterase activities, but not in *Vgsc* gene mutation frequency, suggesting metabolic detoxification mechanism plays a major role in moderate-intensity resistance or high-intensity resistance. Pre-exposure to the PBO synergist restored the susceptibility to insecticide among the highly resistant mosquitoes, confirming the role of monooxygenases in pyrethroid resistance. The rate of resistance decay to be fully susceptible from moderate-intensity resistance took 15 generations, or 15 months under typical tropical Africa climate condition, supporting at least 2-years interval when the rotational use of insecticides with different modes of action is considered for resistance management.

## Data Availability

The datasets used for the current study are available at the repository of the Kenya Medical Research Institute.

## Abbreviations

PCR: Polymerase chain reaction
PBO: Piperonyl butoxide
*Vgsc*: Gene coding for voltage-gated sodium channel
*Ace*-1: Gene coding for acetylcholinesterase-1
GST: Glutathione *S*-transferases
R Group: Populations that were selected for increased pyrethroid resistance
S Group: Populations that were maintained without insecticide selection pressure

## References

[1] Nkya TE, Akhouayri I, Kisinza W, David J-P (2013). Impact of environment on mosquito response to pyrethroid insecticides: Facts, evidences and prospects. Insect Biochem. Mol. Biol..

[2] Ranson H, Lissenden N (2016). Insecticide resistance in African Anopheles mosquitoes: A worsening situation that needs urgent action to mantain malaria control. Parasites Vectors.

[3] WHO. *World Malaria Report 2018*. (World Health Organization, Geneva, 2018).

[4] WHO. *Global Plan for Insecticide Resistance Management.* (World Health Organization, Geneva, 2012).

[5] Reid MC, McKenzie FE (2016). The contribution of agricultural insecticide use to increasing insecticide resistance in African malaria vectors. Malar. J..

[6] Wanjala, C. L. *et al.* Pyrethroid and DDT resistance and organophosphate susceptibility among *Anopheles* spp. mosquitoes from Western Kenya. *Emerg. Infect. Dis.* **21** (2015). 10.3201/eid2112.150814 PMC4672417 26583525

[7] Nkya TE (2014). Impact of agriculture on the selection of insecticide resistance in the malaria vector *Anopheles gambiae*: A multigenerational study in controlled conditions. Parasites Vectors.

[8] Ranson, H. *et al.* Identification of a point mutation in the voltage-gated sodium channel gene of Kenyan *Anopheles gambiae* associated with resistance to DDT and pyrethroids. *Insect Mol. Biol.* **9**, 10.1046/j.1365-2583.2000.00209.x (2000). 10.1046/j.1365-2583.2000.00209.x 11029667

[9] 9Mathias, D. *et al.* Spatial and temporal variation in the kdr allele L1014S in *Anopheles gambiae* s.s. and phenotypic variability in susceptibility to insecticides in Western Kenya. *Malar. J.* **10**, 10.1186/1475-2875-10-10 (2011). 10.1186/1475-2875-10-10 PMC3029224 21235783

[10] Hemming-Schroeder E (2018). Emerging pyrethroid resistance among *Anopheles arabiensis* in Kenya. Am. J. Trop. Med. Hyg..

[11] Ochomo, E. *et al.* Presence of the knockdown resistance mutation, Vgsc-1014F in *Anopheles gambiae* and *An. arabiensis* in western Kenya. *Parasites Vectors* **8**, 616, 10.1186/s13071-015-1223-5 (2015). 10.1186/s13071-015-1223-5 PMC4666190 26626424

[12] Stump, A., Atieli, F., Vulule, J. & Besansky, N. Dynamics of the pyrethroid knockdown resistance allele in western Kenyan populations of *Anopheles gambiae* in response to insecticide-treated bed net trials. *Am. J. Trop. Med. Hyg.* **70** (2004). 15210997

[13] Ochomo, E. *et al.* Pyrethroid resistance in *Anopheles gambiae* s.s. and *Anopheles arabiensis* in western Kenya: Phenotypic, metabolic and target site characterizations of three populations. *Med. Vet. Entomol.* **27**, 156–164, 10.1111/j.1365-2915.2012.01039.x (2012). 10.1111/j.1365-2915.2012.01039.x PMC4744475 22861380

[14] Vulule JM (1999). Elevated oxidase and esterase levels associated with permethrin tolerance in *Anopheles gambiae* from Kenyan villages using permethrin-impregnated nets. Med. Vet. Entomol..

[15] Ingham VA (2020). A sensory appendage protein protects malaria vectors from pyrethroids. Nature.

[16] Yahouédo GA (2017). Contributions of cuticle permeability and enzyme detoxification to pyrethroid resistance in the major malaria vector *Anopheles gambiae*. Sci. Rep..

[17] Lindblade KA (2006). Impact of sustained use of insecticide-treated bednets on malaria vector species distribution and culicine mosquitoes. J. Med. Entomol..

[18] Ranson, H. *et al.* Pyrethroid resistance in African anopheline mosquitoes: What are the implications for malaria control? *Trends Parasitol.* **27**, 10.1016/j.pt.2010.08.004 (2011). 10.1016/j.pt.2010.08.004 20843745

[19] 19Schechtman, H. & Souza, M. O. Costly inheritance and the persistence of insecticide resistance in *Aedes aegypti* populations. *PLoS One* **10** (2015). 10.1371/journal.pone.0123961 PMC4416794 25933383

[20] Scott JA, Brogdon WG, Collins FH (1993). Identification of single specimens of the *Anopheles gambiae* complex by the polymerase chain reaction. Am. J. Trop. Med. Hyg..

[21] Collins FH (1987). A ribosomal RNA gene probe differentiates member species of the *Anopheles gambiae* complex. Am. J. Trop. Med. Hyg..

[22] Shute GT (1956). A method of maintaining colonies of east African strains of *Anopheles gambiae*. Ann. Trop. Med. Parasitol..

[23] WHO. *World Malaria Report 2016*. (World Health Organization, Geneva, 2016).

[24] Bass, C. *et al.* Detection of knockdown resistance (kdr) mutations in *Anopheles gambiae*: A comparison of two new high-throughput assays with existing methods *Malar. J.* **6**, 111 (2007). 10.1186/1475-2875-6-111 PMC1971715 17697325

[25] Jones CM (2012). Footprints of positive selection associated with a mutation (N1575Y) in the voltage-gated sodium channel of *Anopheles gambiae*. Proc. Natl. Acad. Sci..

[26] Bass C, Nikou D, Vontas J, Williamson MS, Field LM (2010). Development of high-throughput real-time PCR assays for the identification of insensitive acetylcholinesterase (ace-1R) in *Anopheles gambiae*. Pestic. Biochem. Physiol..

[27] Brogdon WG, Beach RF, Stewart JM, Castanaza L (1988). Microplate assay analysis of the distribution of organophosphate and carbamate resistance in Guatemalan *Anopheles albimanus*. Bull. World Health Organ..

[28] Zhong D (2013). Relationship between knockdown resistance, metabolic detoxification and organismal resistance to pyrethroids in *Anopheles sinensis*. PLoS ONE.

[29] WHO. *Test Procedures for Insecticide Resistance Monitoring in Malaria Vector Mosquitoes*. (2016).

[30] Core Development Team (2016). R.

[31] Grossman MK (2018). Restoration of pyrethroid susceptibility in a highly resistant *Aedes aegypti* population. Biol. Lett..

[32] Raghavendra K (2010). Persistence of DDT, malathion & deltamethrin resistance in *Anopheles culicifacies* after their sequential withdrawal from indoor residual spraying in Surat district, India. Indian J. Med. Res..

[33] Corbel, V. & N'guessan, R. *Distribution, Mechanisms, Impact and Management of Insecticide Resistance in Malaria Vectors: A Pragmatic Review.**Anopheles Mosquitoes—New Insights into Malaria Vectors. INTECH* (2013).

[34] Shi L (2015). Development of resistance to pyrethroid in *Culex pipiens pallens* population under different insecticide selection pressures. PLoS Negl. Trop. Dis..

[35] Williams J (2019). Characterisation of *Anopheles* strains used for laboratory screening of new vector control products. Parasites Vectors.

[36] Berticat C (2008). Costs and benefits of multiple resistance to insecticides for *Culex quinquefasciatus* mosquitoes. BMC Evol. Biol..

[37] Djihinto AC, Katary A, Prudent P, Vassal J-M, Vaissayre M (2009). Variation in resistance to pyrethroids in *Helicoverpa armigera* from Benin Republic, West Africa. J. Econ. Entomol..

[38] Pwalia R (2019). High insecticide resistance intensity of Anopheles gambiae (sl) and low efficacy of pyrethroid LLINs in Accra, Ghana. Parasites Vectors.

[39] WHO. *World Malaria Report 2018*. (World Health Organisation, Geneva, 2019).

